# Optimizing fluconazole dosing in acute renal failure patients undergoing continuous renal replacement therapy: A population pharmacokinetic/pharmacodynamic study

**DOI:** 10.3389/fphar.2025.1564070

**Published:** 2025-03-28

**Authors:** Shengnan Zhang, Wenhua Zhang, Tingting Wu, Yuanfang Qin, Qi Pei

**Affiliations:** ^1^ Department of Pharmacy, The Third Xiangya Hospital, Central South University, Changsha, China; ^2^ Department of Ophthalmology, The Third Xiangya Hospital, Central South University, Changsha, China

**Keywords:** fluconazole, acute renal failure, continuous renal replacement therapy, population pharmacokinetics, PK/PD, shiny application

## Abstract

Fluconazole pharmacokinetics in acute renal failure (ARF) patients undergoing continuous renal replacement therapy (CRRT) are significantly influenced by the combined effects of impaired renal function and CRRT, yet current dosing guidelines do not account for these complexities, leading to suboptimal therapy and treatment failure. This study aimed to address these limitations by developing a population pharmacokinetic model for fluconazole in ARF patients receiving CRRT, evaluating guideline-recommended dosing regimens for pharmacokinetic/pharmacodynamic target attainment, and then developing software to optimize fluconazole dosing in complex clinical CRRT scenarios. A total of 297 literature-sourced plasma concentration data points from 15 ARF patients and one patient with normal renal function, all receiving CRRT, were used for model construction. The treatment target was set as the 24-h area under the free drug concentration-time curve to the minimum inhibitory concentration ratio ≥100. The web application was developed using R and R packages. The final pharmacokinetic model comprised a central and CRRT compartment, with renal failure and CRRT doses influencing clearance and body weight affecting central compartment distribution volume. Simulations revealed that the guideline-recommended loading (800 mg or 12 mg/kg QD) and maintenance doses (400 mg or 6 mg/kg QD) achieved limited target attainment at low CRRT doses and failed at moderate to high CRRT doses. Consequently, dose adjustments based on body weight and CRRT parameters are recommended. A user-friendly, visual, and interactive Shiny application was developed to assist clinicians in optimizing fluconazole dosing in this challenging patient population.

## 1 Introduction

Invasive fungal disease, mainly caused by opportunistic fungi, imposes a substantial global health burden, with over 150 million severe cases and three million deaths annually (Jenks et al., 2020; Zhang et al., 2023). *Candida* spp. Remain the most common cause of mycoses worldwide, with a mortality rate of up to 50%, largely due to inadequate antifungal therapy (Concia et al., 2009).

Fluconazole, a broad-spectrum triazole antifungal agent, is widely used against *Candida* and *Cryptococcus* species (Pappas et al., 2016; Martin-Loeches et al., 2019). As one of the most commonly used antifungal agents, fluconazole exhibits good pharmacokinetic characteristics and favorable tolerability (Thaler et al., 1995; Charlier et al., 2006). However, significant pharmacokinetic variability of fluconazole has been observed in critically ill patients (Boonstra et al., 2021). Dose optimization based on therapeutic drug monitoring is considered to significantly improve fluconazole exposure in obese, pediatric, and critically ill patients with renal failure (Boonstra et al., 2021; van der Elst et al., 2014; Alobaid et al., 2016).

As a hydrophilic drug with low plasma protein binding (12%) and a molecular weight of 306.2 Da, fluconazole is predominantly excreted unchanged *via* the kidneys (80%) (Debruyne and Ryckelynck, 1993; Roos et al., 2008). In critically ill patients with renal failure, changes in pathological physiological states or organ function can lead to alterations in fluconazole distribution and elimination (Smith et al., 2012). Research has demonstrated that in patients with acute renal failure (ARF), the clearance of fluconazole is reduced to 50% of that in healthy volunteers, with a clearance of 10 mL/kg/h and a half-life period of 96 h, compared to 15–24 mL/kg/h and 30 h in healthy individuals (Bellmann and Smuszkiewicz, 2017; Toon et al., 1990). Moreover, the incorporation of various organ support therapies like renal replacement therapy (RRT) can alter the pharmacokinetics of drugs to varying degrees, with fluconazole being efficiently cleared through the RRT membrane due to its small molecular weight and high water solubility (Bellmann and Smuszkiewicz, 2017; Coenradie et al., 2025). Studies (Oono et al., 1992; Debruyne and Ryckelynck, 1992; Yagasaki et al., 2003; Kishino et al., 2001; Gharibian and Mueller, 2016; Sinnollareddy et al., 2015) have shown that nearly all types of renal replacement therapy clear fluconazole to varying degrees, highlighting its dependence on RRT duration and parameters. This complexity necessitates meticulous consideration of fluconazole pharmacokinetics in critically ill patients undergoing RRT.

Effective antibiotic dosing in critically ill patients is crucial for optimal bactericidal efficacy and clinical outcomes (Kollef et al., 2021), yet current guideline-recommended dosing regimens are considered inadequate for this population (Muilwijk et al., 2020; Pappas et al., 2016; Martin-Loeches et al., 2019). While previous studies have explored fluconazole dose optimization in various critically ill populations, research specific to patients with renal failure on continuous renal replacement therapy (CRRT) remains limited (Boonstra et al., 2021; Alobaid et al., 2016; Muilwijk et al., 2020; Sandaradura et al., 2021; Van Daele et al., 2021). Existing studies, often with small sample size (4–15 patients, 26–80 samples), have not quantitatively assessed the impact of different CRRT types or doses on fluconazole exposure (Muilwijk et al., 2020; Sandaradura et al., 2021; Patel et al., 2011; Novy et al., 2024; Han et al., 2013).

This study aimed to develop a population pharmacokinetic model for critically ill patients undergoing CRRT, quantitatively describe the impact of CRRT on fluconazole clearance, and explore optimal dosing regimens for ARF patients with varying body weights and CRRT doses. Additionally, we developed a model-based fluconazole dose optimization application, aiming to facilitate optimal fluconazole dosing in complex clinical scenarios.

## 2 Materials and methods

### 2.1 Study data collection

**FIGURE 1:**
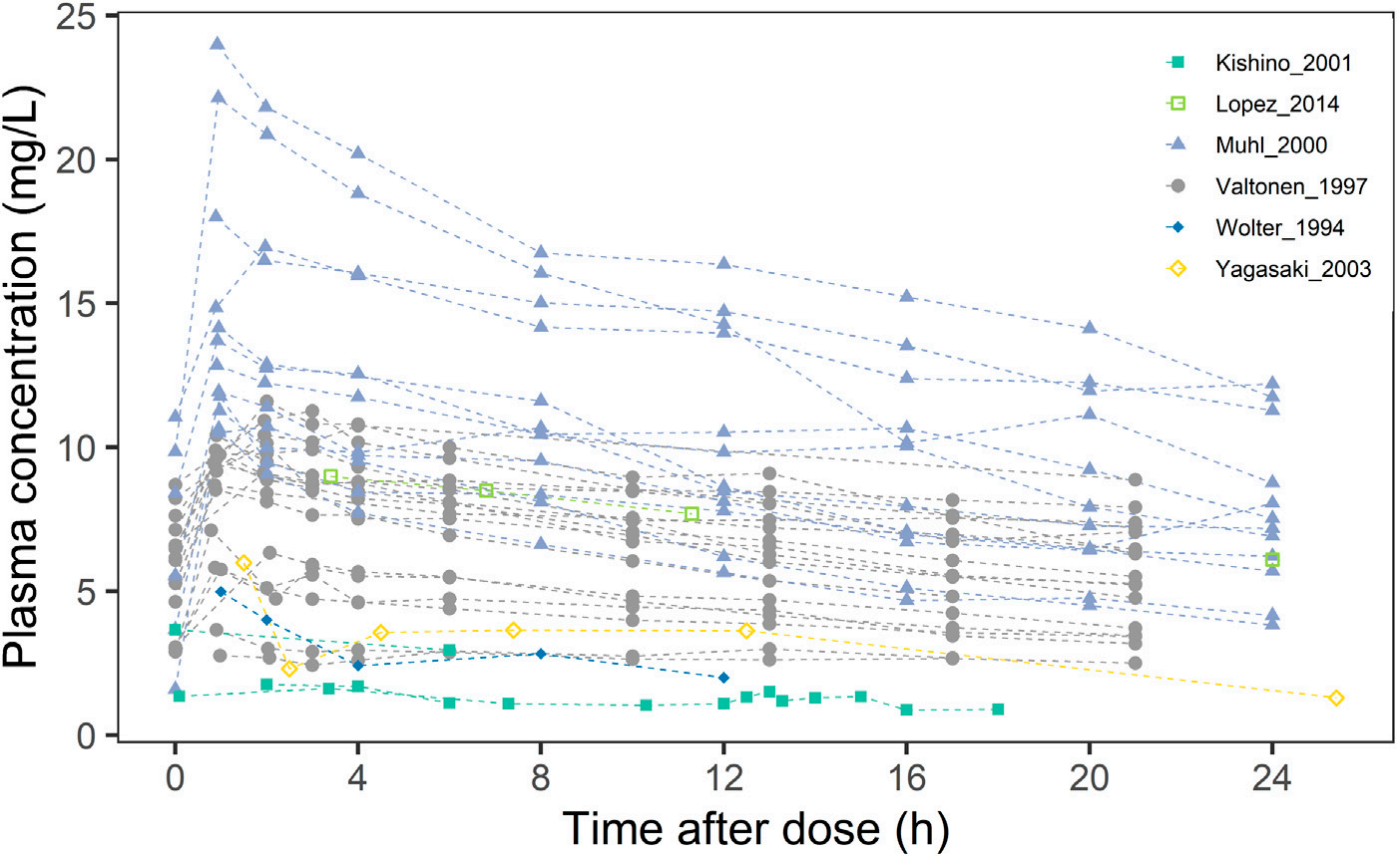
Scatter plot of fluconazole blood concentrations in 16 critically ill CRRT patients from different studies.

A literature search was conducted using the query “((Fluconazole) AND ((((((((continuous renal replacement therapy) OR (CRRT)) OR (continuous venovenous hemofiltration)) OR (continuous venovenous haemodialysis)) OR (continuous venovenous hemodiafiltration)) OR (CVVH)) OR (CVVHD)) OR (CVVHDF))” in the PubMed, Embase and Web of Science databases up to October 2024. Five publications (Yagasaki et al., 2003; Wolter et al., 1994; Valtonen et al., 1997; Muhl et al., 2000; Lopez and Phillips, 2014) were identified based on predefined inclusion criteria, including the availability of individual plasma concentration-time data, specific dosing information, sampling schemes, and sufficient CRRT parameter details for model development. Data on plasma concentration and accumulated drug dosage in the filtrate were digitized using WebPlotDigitizer (version 4.3). The detailed patient demographics, fluconazole doses, and CRRT parameter settings were summarized in Table 1. Missing values were imputed with mean or median values. Based on CRRT mode and relevant parameters, we calculated CRRT clearance, which were detailed in the Supplementary Material (Section 1). A scatter plot of plasma concentration over time was shown in Figure 1.

**TABLE 1:** Summary of demographics, dosage regimens, and CRRT parameters for included studies.

Characteristics	Wolter et al. [Wolter et al., 1994]	Valtonen et al. [Valtonen et al., 1997]	Muhl et al. [Muhl et al., 2000]	Kishino et al. [Kishino et al., 2001]	Yagasaki et al. [Yagasaki et al., 2003]	Lopez et al. [Lopez and Phillips, 2014]	Overall
Demographics							
Male/Female	0/1	4/2	3/3	0/1	1/0	1/0	9/7
Age^a^	61	62.5 (32–72)	NA (61–82)	57	70	48	59.5 (32–82)
Body weight (kg)^a^	76	81.5 (72–125)	72.5 (55–165)	48	54	272	77 (48–272)
ARF	Yes	Yes	Yes	No	Yes	Yes	15/1
Dose regimens							
Dose (mg)	200	200	400–800	50–200	200	600–1,200	50–1,200
Infusion time (h)	0.5	1	1–2	1	1	3–6	0.5–6
CRRT parameters							
CRRT type	CVVHD	CVVH CVVHDF	CVVH CVVHDF	CVVHDF	CVVH	CVVHDF	13/1/14
Q_b_ (mL/min)	75	100	90	100	105	180	75–180
CRRT dose (mL/h/kg)	13.2	8.0–41.7	6.1–36.4	62.5	64.8	8.8	8.0–64.8
S_c_/S_d_ ^a^	0.67	0.49 (0.26–0.71)	0.87 (0.54–1.02)	0.67	0.67	0.67	0.67

ARF, acute renal failure; CRRT, continuous renal replacement therapy; Q_b_, blood flow rate; S_c_/S_d_, sieving coefficient/saturation coefficient; CVVH, continuous veno-venous hemofiltration; CVVHD, continuous veno-venous hemodialysis; CVVHDF, refers to continuous veno-venous hemodiafiltration; NA, not applicable.

^a^ indicate the median, with the numbers in parentheses representing the minimum and maximum values.

### 2.2 Population PK modelling

A nonlinear mixed effects modelling approach was undertaken using NONMEM^®^ software (version 7.3, Icon Development Solutions, Ellicott City, MD, USA), with parameter estimation executed *via* the FOCEI algorithm. The Perl-Speaks-NONMEM program (version 4.60, Uppsala University, Uppsala, Sweden) supported the modelling processes, while Pirana software (version 2.9.6, Pirana Software & Consulting BV) served as the interface. Analysis and visualization of the NONMEM output were accomplished using the R package (version 4.3.1; http://www.r-project.org).

**FIGURE 2:**
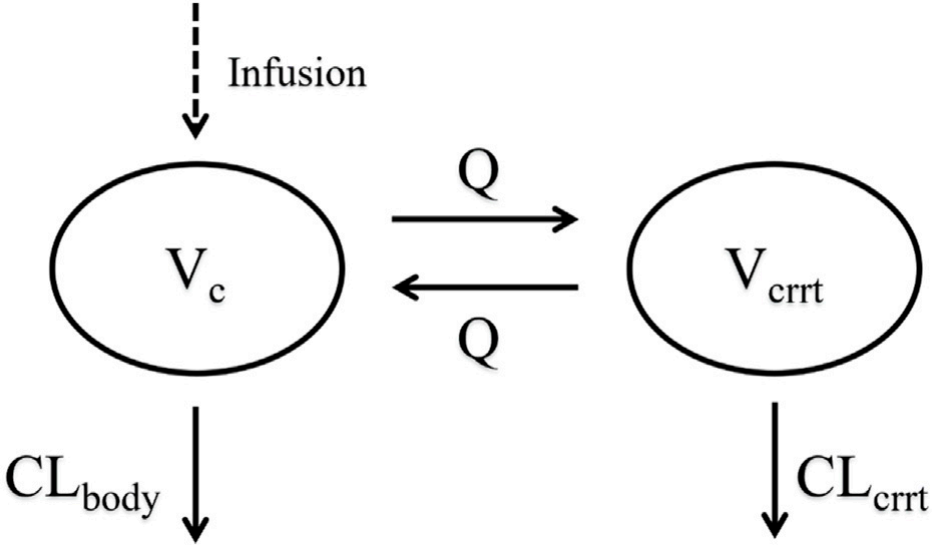
Schematic diagram of the model structure. CL_body_, central compartment clearance; CL_crrt_, CRRT compartment clearance; V_c_, central compartment apparent distribution volume; Q, intercompartmental clearance; V_crrt_, the apparent distribution volume of the CRRT compartment.

Structural models, including one-compartment and two-compartment models, as well as one-compartment models with a tandem CRRT compartment, were evaluated. The selection of the structural model was based on comparisons of the objective function value and goodness of fit (GOF) plots. Different random effects models were assessed separately, with preferences given to models exhibited the smallest variance values. The final structural model was a one-compartment model incorporating a CRRT compartment in series with total clearance (CL_Total_) calculated as the sum of CRRT clearance (CL_crrt_) and residual body clearance (CL_body_). The final model structure was shown in Figure 2.

Covariate modelling was employed to delineate interindividual variation in pharmacokinetic parameters. Potential covariates such as age, body weight, and filter membrane area were considered continuous factors, while sex, ARF, and filter membrane type were regarded as categorical variables. Covariates were identified using a stepwise method comprising forward inclusion and backwards elimination.

The model was assessed using GOF, prediction-corrected visual predictive check (pc-VPC), and sampling importance resampling (SIR) analyses. GOF plots facilitated visual comparison between predicted and observed values, including the distribution and trend of residuals. Pc-VPC, a simulation-based diagnostic tool, utilizes 1,000 simulations to examine model prediction performance. SIR analyses employed 1,000 final proposal samples and 1,000 resamples to evaluate the uncertainty of the parameter estimates.

### 2.3 PK/PD analysis using Monte Carlo simulations

Monte Carlo simulations assessed the probability of pharmacokinetic/pharmacodynamic (PK/PD) target attainment (PTA) for various fluconazole dosing regimens in patients with different body weights and CRRT clearance intensities (n = 1,000). The PK/PD target was defined as the ratio of the plasma-free fluconazole AUC to the minimum inhibitory concentration (*f*AUC/MIC), with a threshold of 100 according to the European Committee on Antimicrobial Susceptibility Testing (EUCAST) (EUCAST, 2020b). Based on the primary MIC distribution of *Candida* species to fluconazole, the MICs ranging from 0.06–32 mg/L were examined, with particular attention given to the clinical susceptibility breakpoints of 2 mg/L defined by the EUCAST and 4 mg/L (susceptible dose-dependent breakpoint) defined by the Clinical and Laboratory Standards Institute (CLSI) (EUCAST, 2020a; Pappas et al., 2016). The simulations assumed fluconazole plasma protein binding at 12% and utilized mean sieving coefficients to calculate CRRT clearance.

To explore optimal efficacy, six fixed-dose regimens (200 mg, 400 mg, 600 mg, 800 mg, 1,000 mg, and 1,200 mg QD) and four weight-based dosing regimens (6 mg/kg, 8 mg/kg, 10 mg/kg, and 12 mg/kg QD) were examined across four body weight tiers (45 kg, 70 kg, 95 kg, and 120 kg) and four CRRT doses (20 mL/kg/h, 35 mL/kg/h, and 50 mL/kg/h). The PTAs were calculated for the various loading and maintenance doses on the first day and at a steady state. Any dosing regimen achieving a PTA ≥90% was considered optimal.

### 2.4 Development of a shiny application for fluconazole dose optimization

An interactive R-based application was developed using software packages such as Shiny and Mrgsolve, with the final model embedded. The input data included patient information, dosing regimen (loading dose, maintenance dose, frequency, and supplemental dosing), RRT-related information (start and end times, intensity of each RRT), and microbial drug sensitivity information. Supplemental dosing scenarios specify the additional dose to be administered during each RRT session or at the next dose after RRT ends.

## 3 Results

### 3.1 Study population and pharmacokinetic data

The population pharmacokinetic analysis included 16 patients undergoing CRRT (15 ARF patients and one liver transplant patient) from five published studies. Patient characteristics were detailed in Table 1, with nine males in the cohort. The median age and weight were 72 years and 77 kg, respectively. Among the renal failure patients, seven were anuric, and seven were oliguric. The fluconazole dosages ranged from 50–1,200 mg.

### 3.2 Population PK modelling

**FIGURE 3:**
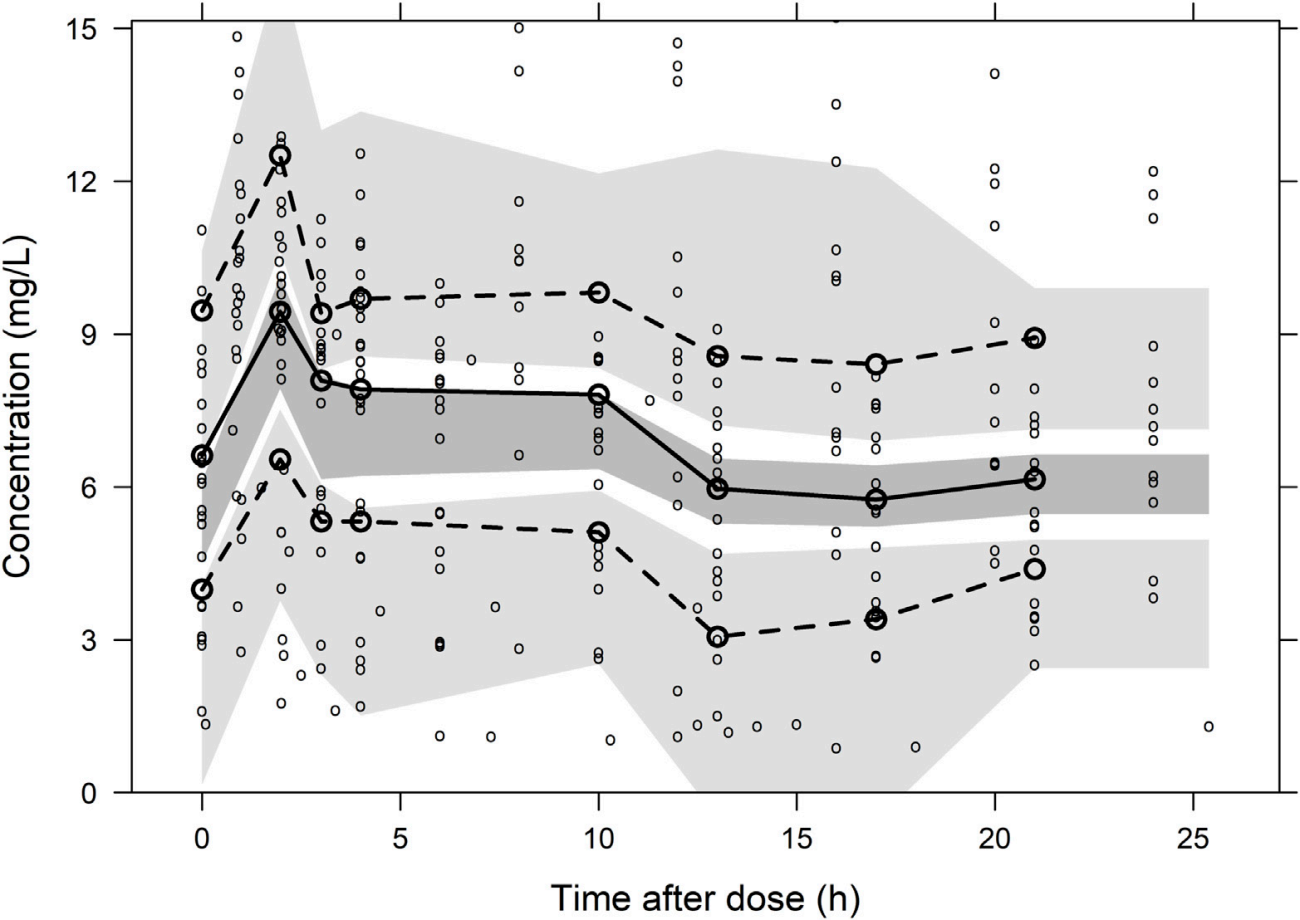
Prediction-corrected visual predictive check plot for the final population pharmacokinetic model of Fluconazole. The lower, middle and upper solid lines are the 5%, 50% and 95% quantile lines of the observed concentrations, and the shaded areas represent the 95% confidence intervals for the prediction lines.

A total of 297 plasma concentration points from 16 patients were utilized for population pharmacokinetic modelling. The model structure, comprising a central compartment in tandem with the CRRT compartment, best fits the plasma concentration-time data. The central compartment describes the body’s drug clearance, while the CRRT compartment describes drug elimination *via* CRRT. Drug transfer between these compartments is denoted by Q. The residual model employed a summed residual structure. Covariate analysis incorporated the presence of renal failure for clearance and total body weight for volume of distribution. Table 2 summarizes the final model parameters and associated uncertainties. The relative standard error and SIR results confirmed the accurate estimation of the PK parameters. The GOF plots demonstrated a good fit of the model predictions to the observed data, with a symmetrical residual distribution (Supplementary Material, Supplementary Figure S2). The pc-VPC showed good predictive performance of the final model (Figure 3).

**TABLE 2:** Estimated values of fluconazole final model parameters and SIR validation results.

Parameters	Final PK model		SIR-results^a^		Shrinkage %
	Estimate	RSE%	Median	95% CI	
Fixed effect parameters					
CLbody _arf (L/h)	0.41	15%	0.41	0.30–0.52	-
CLbody _nrf (L/h)	1.25	23%	1.22	0.69–1.81	-
Vc (L)	37.90	11%	38.06	30.65–45.27	-
Vcrrt (L)	23.50	12%	23.57	18.21–28.82	-
Q (L/h)	38.80	25%	37.04	20.07–48.95	-
WT on Vc	0.799	7%	0.802	0.690–0.914	-
Interindividual variability (ω2)					
CL	0.187	18%	0.179	0.054–0.297	15.9
Vc	0.097	29%	0.087	0.016–0.169	7.8
Vcrrt	0.084	59%	0.102	0.006–0.255	36.4
Q	0.412	35%	0.378	0.027–0.845	44.5
Residual variability (σ)					
Additive error (mg/L)	0.375	13%	0.383	0.298–0.473	5.93

SIR, sampling importance resampling; RSE, relative squared error; 95% CI, 95% confidence interval; CLbody _arf, fluconazole body clearance in patients with acute renal failure; CLbody _nrf, fluconazole body clearance in patients with normal renal function; Vc, distribution volume of central compartment; Vcrrt, distribution volume of CRRT, compartment; Q, intercompartmental clearance; WT, weight.

^a^ SIR, procedure was executed with 1,000 final proposal samples and 1,000 resamples.

### 3.3 Simulation-based PK/PD analysis

**FIGURE 4:**
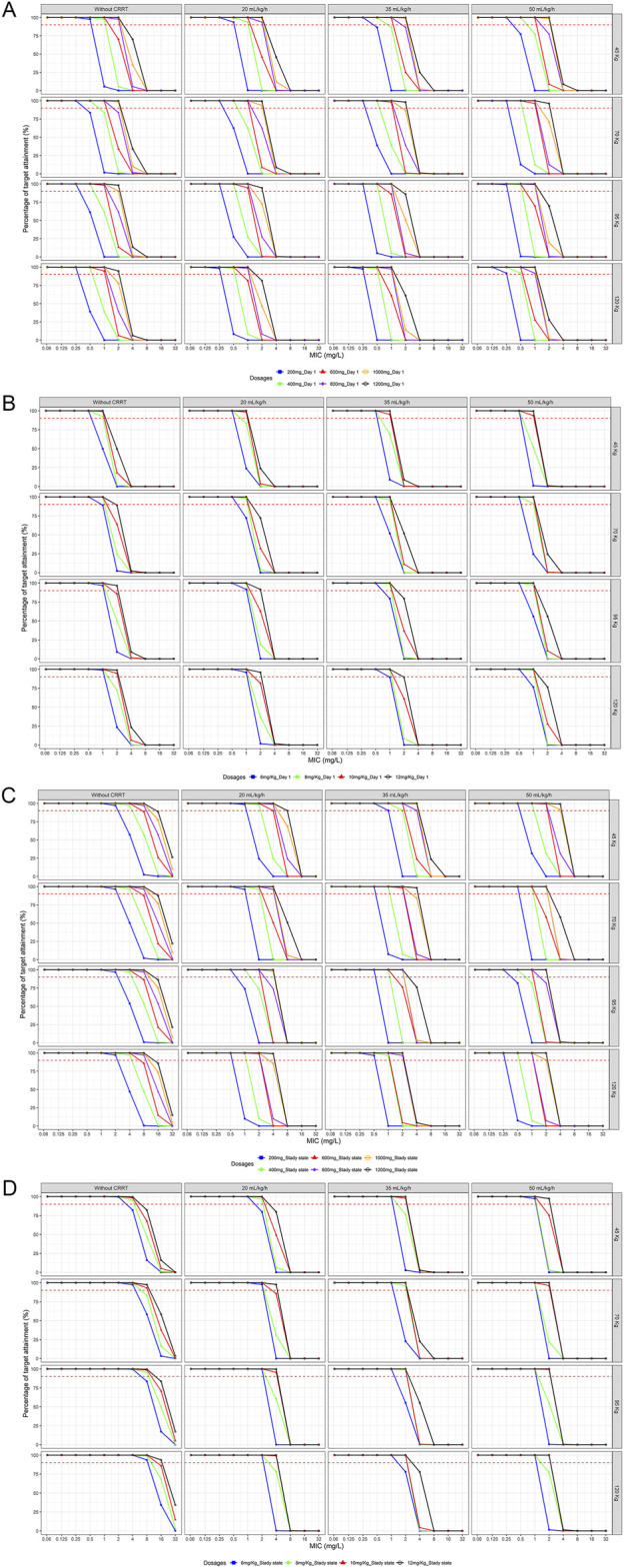
Probability of target attainment of various loading and maintenance dose regimens for different body weights and CRRT doses **(A)** Fixd-dose regimens at Day 1 **(B)** Weight-based dosing regimens at Day 1 **(C)** Fixd-dose regimens at steady state **(D)** Weight-based dosing regimens at steady state (PK/PD target: *f*AUC/MIC≥100)

The simulations indicated that loading doses of 800 mg, 1,000 mg, 12 mg/kg and 12 mg/kg were necessary to achieve 90% PTA on day 1 (Figures 4A,B) for ARF patients across four different weight groups, and were slightly lower for patients without CRRT. For fungi with an MIC of 4 mg/L, none of the tested dosing regimens met the target on day one.

Upon reaching a steady state (Figures 4C,D), a 200 mg maintenance dose was effective against fungi with MIC ≤2 mg/L in patients without CRRT. Even in patients with higher body weights (120 kg), a maintenance dose of 400 mg achieved optimal targets against fungi with an MIC of 4 mg/L. The maintenance dose required for ARF patients varied significantly across different body weights and CRRT intensities. None of the maintenance dose regimens tested at medium to high body weights or CRRT doses met the target for fungi with an MIC ≥4 mg/L.

Regarding the guideline-recommended loading doses of 800 mg and 12 mg/kg for *Candida* infections (Pappas et al., 2016; Martin-Loeches et al., 2019), simulations suggested their appropriateness for ARF patients without RRT or with low-flow CRRT, ensuring good PD coverage against fungi with an MIC ≤2 mg/L. When CRRT clearance rates reached 35 mL/kg/h, higher loading doses were indicated. Among the commonly used maintenance dose regimens of fluconazole in critically ill patients, a 200 mg maintenance dose was found to be effective against fungi, with an MIC of 1 mg/L primarily at lower body weights and CRRT flow rates. The 400 mg maintenance dose was effective against fungi with an MIC of 2 mg/L in lower weight scenarios (e.g., 45 kg with 35 mL/kg/h or 70 kg with 20 mL/kg/h). The weight-based 6 mg/kg maintenance dose regimen was effective only in patients with lower CRRT flow rates (70–120 kg, mL/kg/h 20 mL/kg/h) against fungi with an MIC of 2 mg/L. For fungi with an MIC of 4 mg/L, only the 400 mg maintenance dose regimen met the 90% threshold in ARF patients without RRT. The detailed minimum attainment doses were listed in Tables 3 and 4.

**TABLE 3:** Minimum target attainment loading and maintenance dose regimens when MIC = 2 mg/L.

	45 Kg		70 Kg		95 Kg		120 Kg	
	LD (mg)	MD (mg)	LD (mg)	MD (mg)	LD (mg)	MD (mg)	LD (mg)	MD (mg)
0 mL/kg/h	800	200	840	200	1,000	200	1,200	200
20 mL/kg/h	800	360	1,000	400	1,140	570	1,440	600
35 mL/kg/h	1,000	400	1,200	560	--	760	--	800
50 mL/kg/h	1,000	540	1,200	700	--	950	--	1,000

LD, loading dose; MD, maintenance dose; “--” means none of the regimens met the target; PK/PD, target: *f*AUC/MIC≥100.

**TABLE 4:** Minimum target attainment maintenance dose regimens when MIC = 4 mg/L.

	45 Kg	70 Kg	95 Kg	120 Kg
	MD (mg)	MD (mg)	MD (mg)	MD (mg)
0 mL/kg/h	360	400	400	400
20 mL/kg/h	600	800	950	1,200
35 mL/kg/h	800	1,200	--	--
50 mL/kg/h	1,000	--	--	--

LD, loading dose; MD, maintenance dose; None of the loading dose regimens met the target at first day. PK/PD target: *f*AUC/MIC≥100.

### 3.4 Interactive shiny application for fluconazole dose optimization

**FIGURE 5:**
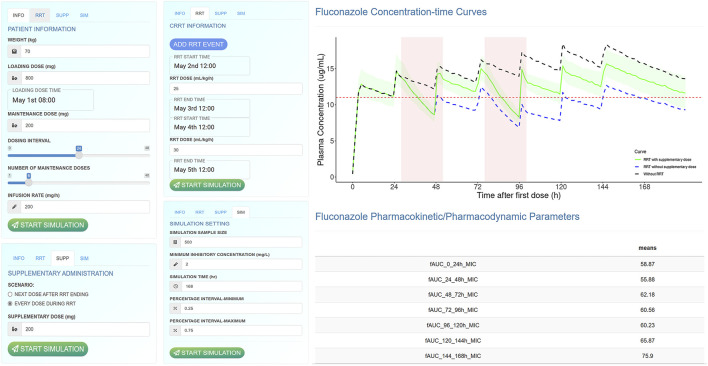
The interface of fluconazole dose optimization shiny application.

A user-friendly R Shiny application for fluconazole dose optimization in ARF patients receiving RRT was developed and is freely available at https://xy3yx.shinyapps.io/fluconazole-crrt-dosing/, with the interface shown in Figure 5 and a detailed user guide in the Supplementary Material (Supplementary Material, Section 4). The application integrates patient information, dosing details (including whether supplemental dosing was performed and its timing), and RRT-related information. It performs Monte Carlo simulations based on the final model, visualizing the predicted results as plasma concentration-time curves and *f*AUC/MIC values every 24 h during the treatment period, and updating them in real-time. It allows for the simulation of multiple intermittent RRT sessions with and without supplemental dosing, highlighting the differences in PK/PD results. Additionally, the application features a user-friendly point-and-click interface for time information input, allowing users to enter actual dosing and RRT times down to the minute. This approach enhances accuracy and flexibility, reducing the physician’s computational workload.

## 4 Discussion

Fluconazole exhibits significant pharmacokinetic variability in critically ill populations (Boonstra et al., 2021; Muilwijk et al., 2020; Sandaradura et al., 2021), particularly those with renal failure, where clearance is often dependent on RRT. Previous research showed that RRT enhances fluconazole clearance in a dose-dependent manner (Bellmann and Smuszkiewicz, 2017; Valtonen et al., 1997; Muhl et al., 2000), complicating its pharmacokinetics in ARF patients on RRT. This study developed the largest population pharmacokinetic model of fluconazole in ARF patients undergoing CRRT to date, which provides valuable insights into the impact of CRRT dose on optimal fluconazole dosing in this population.

In contrast to previous fluconazole pharmacokinetic models (Muilwijk et al., 2020; Sandaradura et al., 2021; Patel et al., 2011; Novy et al., 2024; Han et al., 2013), our model structure combined a central compartment and a CRRT compartment, effectively characterizing the pharmacokinetic dataset. We posit that this model aligns more closely with the clearance patterns of fluconazole among CRRT patients. Our model showed good consistency with previous findings, estimating residual clearance in ARF patients at 0.407 L/h (Toon et al., 1990; Muilwijk et al., 2020; Sandaradura et al., 2021; Han et al., 2013), whole body clearance at 1.25 L/h in non-ARF patients (Debruyne and Ryckelynck, 1993), and central compartment distribution volume (Vc) of 37.9 L, indicating the reliability of the model (Muilwijk et al., 2020; Patel et al., 2011). For obese critically ill patients, weight-based dosing regimens were recently advocated (Alobaid et al., 2016). Previous studies (Sandaradura et al., 2021; Novy et al., 2024) have incorporated the impact of body weight on CL or Vc. In our study, the body weight-to-70 kg ratio was integrated into the Vc calculation using a power function. In this study, although we had 297 samples, they were derived from only 16 patients. The SIR method was chosen for parameter uncertainty estimation due to its robustness in small sample size scenarios compared to the bootstrap (Dosne et al., 2016).

Simulations revealed that ARF patients require different maintenance doses based on CRRT status and dose. While guideline-recommended loading doses (800 mg or 12 mg/kg QD) and maintenance dose regimens (400 mg or 6 mg/kg QD) for *Candida* infections were effective against fungi with an MIC ≤1 mg/L, they only partially met the current susceptibility breakpoints for *Candida* (4 mg/L by CLSI and 2 mg/L by EUCAST) in patients not receiving RRT or under low CRRT flow. These findings were consistent with prior research (Boonstra et al., 2021; Muilwijk et al., 2020; Sandaradura et al., 2021). Sandaradura et al. (Sandaradura et al., 2021) recommended a loading dose of 800 mg for critically ill underweight (40 kg) patients and a 12 mg/kg loading dose for patients of other body weights. Boonstra et al. (Boonstra et al., 2021) suggested that critically ill adults not undergoing CRRT require a loading dose of either 1,000 mg or 12 mg/kg for fungi with an MIC of 2 mg/L. In our study, 45 kg patients required at least an 800 mg loading dose, and patients with other body weights could be treated with either a 1,000 mg or 12 mg/kg initial dosing regimen. For fungi with an MIC of 4 mg/L, Muilwijk et al. (Muilwijk et al., 2020) advocated a maintenance dose of 400 mg for patients with impaired renal function and 800 mg for those undergoing CRRT. Our findings suggest that typical weight ARF patients (70 kg) necessitate approximately the same maintenance dose without RRT or at low CRRT flow rates, whereas a CRRT dose of 35 mL/kg/h mandates a maintenance dose of 1,200 mg or higher. Notably, most *Candida* species remain highly susceptible to fluconazole *in vitro*. The MIC_90_ values for *C. albicans*, *C. parapsilosis*, and *C. tropicalis* were 0.5, 2, and 2 mg/L, respectively, with only 3%, 8%, and 10% of the isolates having an MIC ≥4 mg/L (EUCAST, 2020b).

We developed an interactive web-based dose optimization application using R, which allows for a highly flexible input of renal replacement therapy events, enabling physicians to directly input actual or anticipated usage times and dialysis intensity. Additionally, this Shiny-based dose optimization application allows for the precise input of detailed timing (including date and time) and specific RRT information (including time, intensity, and frequency). Furthermore, we incorporated two possible supplementary dosing scenarios to enable physicians to promptly correct low drug concentrations. The real-time update of the PK/PD results output promotes the selection of the optimal dosing regimen.

Our study has several limitations, including reliance on digitized concentration data and literature-derived dosing and CRRT information, which may introduce discrepancies between the obtained data and the actual data. However, digitization methods have been demonstrated to effectively generate accurate data in previous studies (Wojtyniak et al., 2020) and have been confirmed in our application as well (Supplementary Material, Section 1). Another major limitation was the insufficient external validation due to the limited availability of data. Although 297 concentration data points were utilized, they were derived from only 16 patients, which may constrain the generalizability of the findings to a broader patient population. The model has been primarily validated in only a small number of samples from two studies (Patel et al., 2011; Sinnollareddy et al., 2015) (Supplementary Material, Section 3). Future prospective studies are required to further validate the accuracy and effectiveness of both the model and the software.

## 5 Conclusion

This study successfully established a population pharmacokinetic model of fluconazole in ARF patients with CRRT based on literature-sourced concentration data, revealing that guideline-recommended dosing regimens may be insufficient under moderate to high CRRT doses. Dosing adjustments based on body weight and CRRT dose are recommended to achieve optimal therapeutic targets. The developed R Shiny application provides a practical tool for clinicians to optimize fluconazole dosing in complex clinical settings.

## Data Availability

The original contributions presented in the study are included in the article/Supplementary Material. Further inquiries can be directed to the corresponding authors.
