# Supplementary material for: Optimizing fluconazole dosing in acute renal failure patients undergoing continuous renal replacement therapy: A population pharmacokinetic/pharmacodynamic study
Source: Front Pharmacol. 2025 Mar 28;16:1564070. doi: 10.3389/fphar.2025.1564070 (PMC11985842; doi:10.3389/fphar.2025.1564070)
Supplement: Supplementary file 1 [file DataSheet1.docx]

**Supplementary Material**

**Section one: Digital software verification and calculation of CRRT clearance**

To verify the precision of the data extracted via the digital software, we compared the raw concentration data ([Aoki et al., 2018](#_ENREF_1)) with the digitized data obtained from the literature graphs (Table S1). This evaluation involved analyzing the distribution of concentration points within the 95% consistency boundaries of Bland-Altman plots. As illustrated in Figure S1, the mean difference between the measured and extracted concentrations was 0.0004 mg/L, with 95% consistency limits ranging from -0.001764 to 0.002544 mg/L. All data points fell within these intervals, demonstrating that the concentrations extracted using the digital software were consistent with the actual measurements and reliable for extracting concentration data from literature sources.

**Table S1** Comparison of the raw and extracted data.

| Day | Complicated (µg/L) | | Uncomplicated (µg/L) | |
| --- | --- | --- | --- | --- |
|  | Raw data | extracted data | Raw data | extracted data |
| 1 | 0.5017 | 0.5000 | 0.1703 | 0.1700 |
| 2 | 0.5109 | 0.5100 | 0.1703 | 0.1700 |
| 3 | 0.4486 | 0.4500 | 0.1491 | 0.1500 |
| 5 | 0.2319 | 0.2300 | 0.0893 | 0.0900 |
| 7 | 0.1310 | 0.1300 | 0.1703 | 0.1700 |


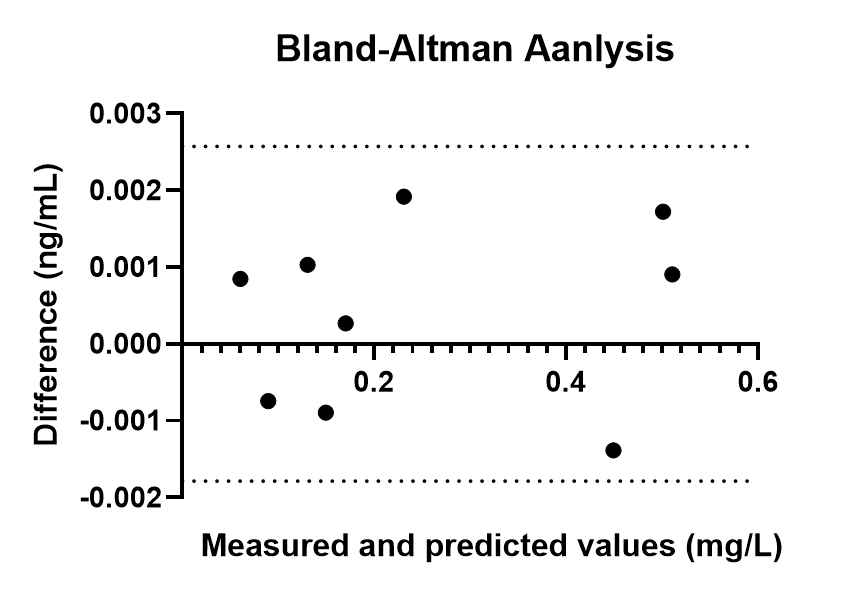


**Figure S1** Bland‒Altman analysis of the digitally extracted data vs. the raw data

**The calculation of CRRT clearance**

The calculation of CRRT clearance depends on the CRRT mode used, as well as relevant parameters such as the ultrafiltrate flow rate, dialysate flow rate, and sieving/saturation coefficients. The CRRT clearance for each patient was computed using the following equation:

CVVH mode：CL_CRRT_ = Q_uf_ * S_c_

CVVHD mode：CL_CRRT_ = Q_d_ * S_d_

CVVHDF mode：CL_CRRT_ = (Q_uf_ + Q_d_) * S_d_

, where Q_uf_ is the ultrafiltration flow rate (L/h), Q_d_ is the dialysate flow rate (L/h), Q_b_ is the blood flow rate (L/h), S_c_ is the sieving coefficient, and S_d_ is the saturation coefficient. S_c_ or S_d_ were derived from the area under the time curve of drug concentration in the filtrate versus the area under the time curve of plasma drug concentration (AUC_f_/AUC_p_) ([Muhl et al., 2000](#_ENREF_2)), with any missing data imputed using mean values. Total clearance (CL_Total_) was defined as the sum of CRRT clearance and residual clearance: CL_Total_ = CL_body_ + CL_CRRT_

**Section two: The NONMEM code**

$PROBLEM Fluconazole CRRT

$DATA fluconazole.csv IGNORE=#

$INPUT ID GENDER AGE WT URINE AMT TIME TAD RATE DV MDV EVID CRRTTYPE CRRTYN MRMTYPE MEMAREA MS CLCRRT

$SUBROUTINES ADVAN6 TOL=3

$MODEL NCOMP=2

COMP= (CENTRAL, DEFDOS, DEFOBS)

COMP=(CRRT)

$PK

CL= THETA (1) *EXP (ETA (1))

IF (URINE.EQ.4) CL =THETA (2) *EXP (ETA (1))

IF (CRRTYN.EQ.0) CL_crrt = 0

IF (CRRTYN.EQ.1) CL_crrt = CLCRRT

V1=THETA (3) *(WT/70) **THETA (6) *EXP (ETA (2))

V2=THETA (4) *EXP (ETA (3))

Q=THETA (5) *EXP (ETA (4))

S1=V1

S2=V2

K10=CL/V1

K12=Q/V1

K21=Q/V2

K20=CL_crrt/V2

$DES

DADT (1) = -K10*A (1) -K12*A (1) + K21* A (2)

DADT (2) = K12*A (1) - K20*A (2) - K21*A (2)

$ERROR

IPRED=F

W=F

IRES=DV-IPRED

IWRES=IRES/F

Y=F+EPS (1)

$THETA

(0,0.4,0.8); CL_arf

(0,1.25,2); CLnrf

(0,42,60); Vc

(0,22,40); Vcrrt

(0,36,50); Q

(0,0.8,1); WT-Vc

$OMEGA

0.2; CL

0.1; Vc

0.1; Vcrrt

0.3; Q

$SIGMA

1; RUV-1

$ESTIMATION METHOD=1 INTER MAXEVAL=9999 PRINT=20 NOABORT

$COVARIANCE PRINT=E

**Section Three: Model Performance Evaluation and External Validation**


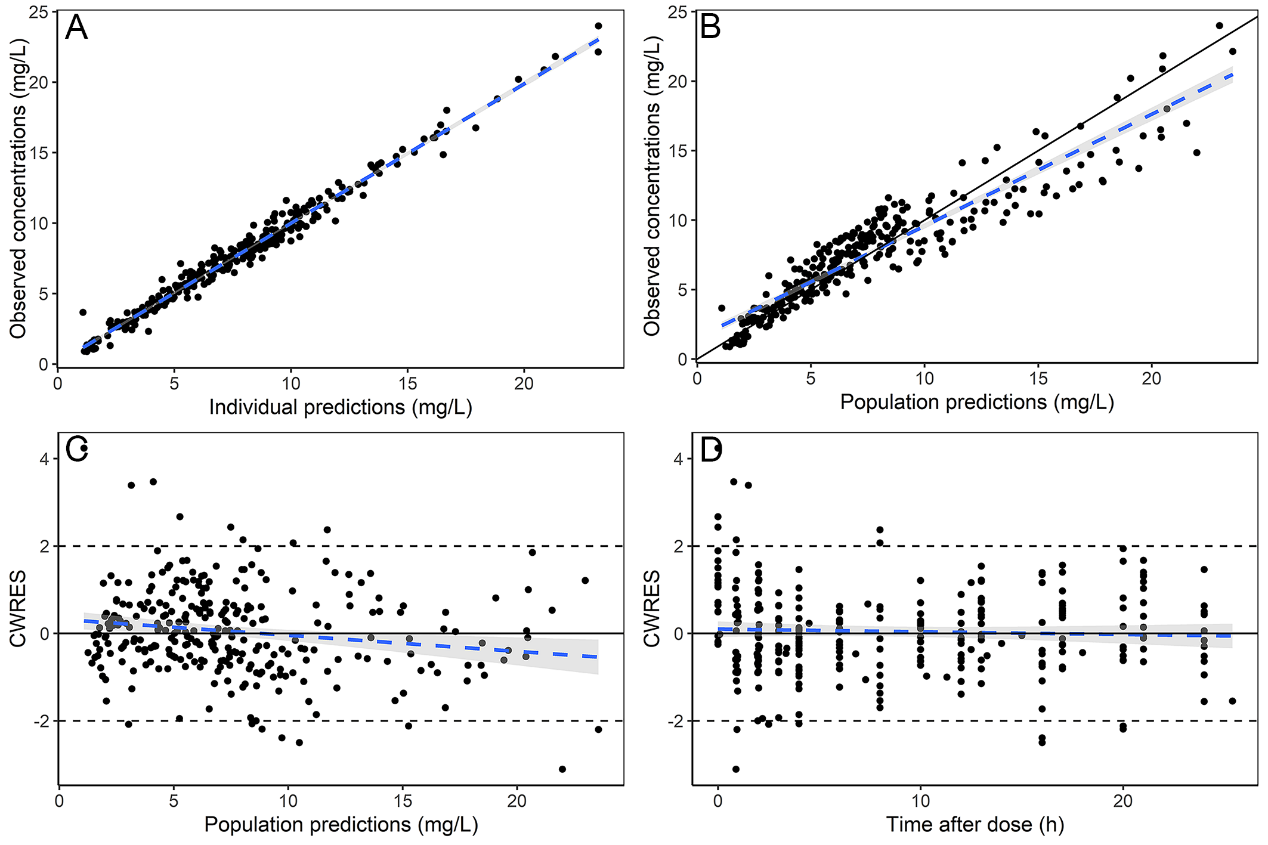


**Figure S2** Goodness-of-fit plots of the final fluconazole population pharmacokinetic model. A: observed concentrations versus individual predictions of fluconazole in plasma. B: observed concentrations versus population predictions of fluconazole in plasma. C: conditional weighted residuals (CWRES) versus population predicted fluconazole concentrations. D: CWRES versus Time after dose.


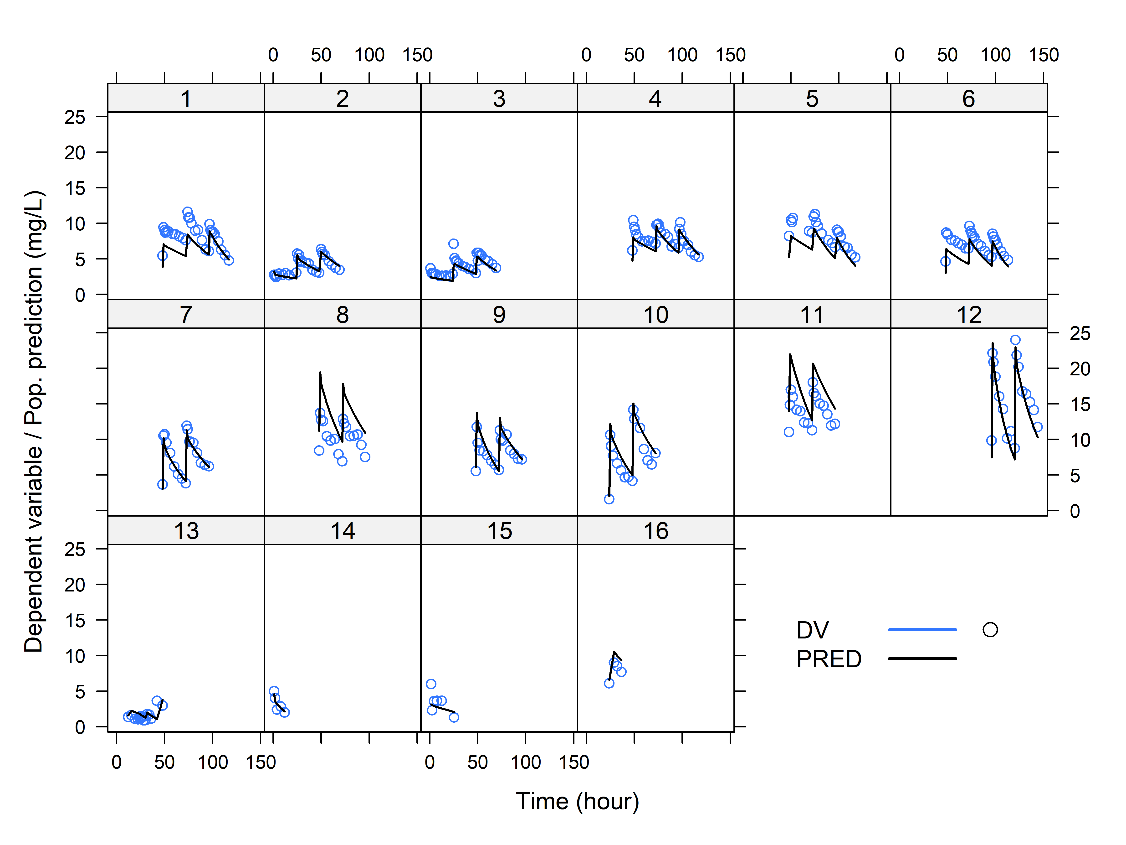


**Figure S3** Individual data fitting plot showing observed plasma concentrations and population-predicted values for each subject.


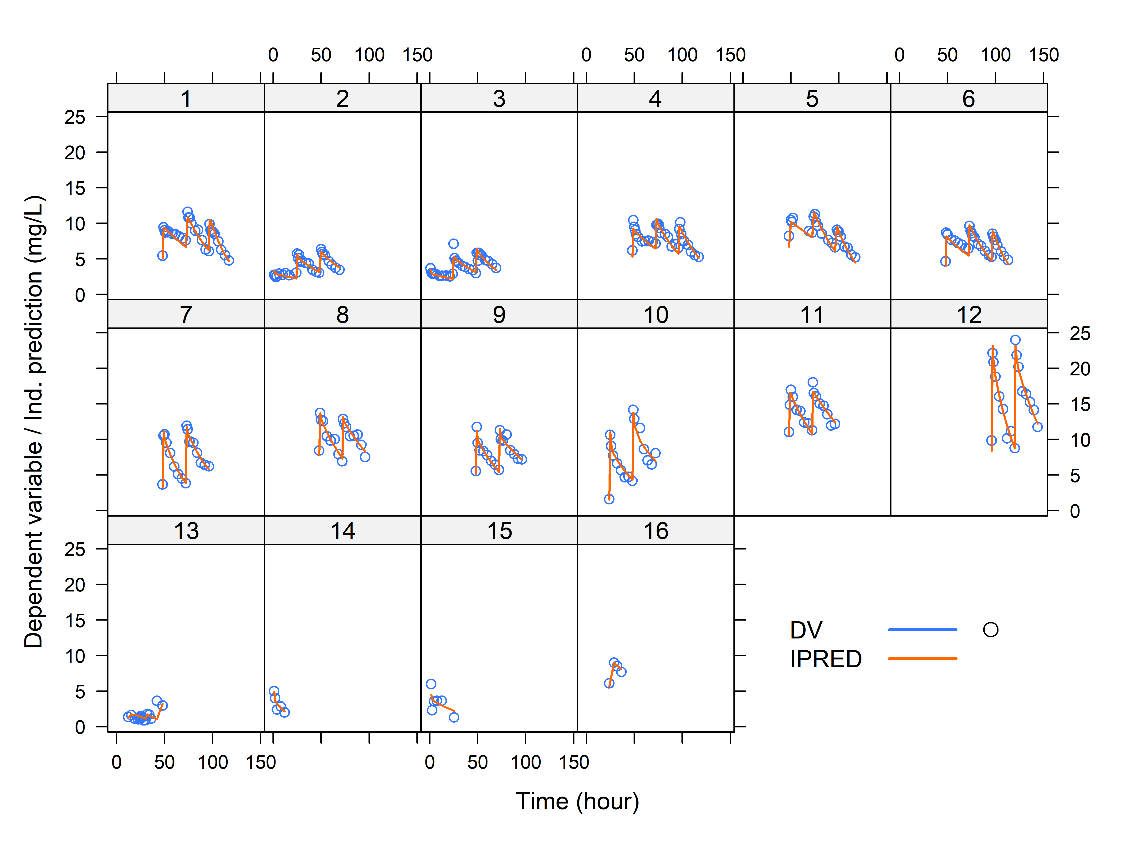


**Figure S4** Individual data fitting plot showing observed plasma concentrations and individual-predicted values for each subject.

We conducted a preliminary external validation using 20 plasma concentration points from two studies ([Patel et al., 2011](#_ENREF_3), [Sinnollareddy et al., 2015a](#_ENREF_5)) that were not included in the original model development. Another relevant study was excluded from the validation process due to the unavailability of specific RRT protocols ([Sinnollareddy et al., 2015b](#_ENREF_6)). The validation results indicated that the observed values fell within the 90% prediction intervals of the model and were relatively evenly distributed around the median pharmacokinetic curves, indicating good predictive performance of the model.

**Table S2** External validation data

| ID | Dosing regimen | | | Renal replacement therapy | | |
| --- | --- | --- | --- | --- | --- | --- |
|  | Dose  (mg) | Frequency | Days of Dosing | Q_UF_  (L/h) | Q_D_  (L/h) | Days of RRT |
| 1^a^ | 200 | qd | 10 | 3.96 | 12 | 5 |
| 2^b^ | 200 | bid | 1 | 2 | 1 | 0.5 |

a, reference ([Sinnollareddy et al., 2015a](#_ENREF_5)); b, reference ([Patel et al., 2011](#_ENREF_3)); Q_UF_, ultrafiltration flow rate (L/h); Q_D_, dialysate flow rate (L/h); RRT, renal replacement therapy


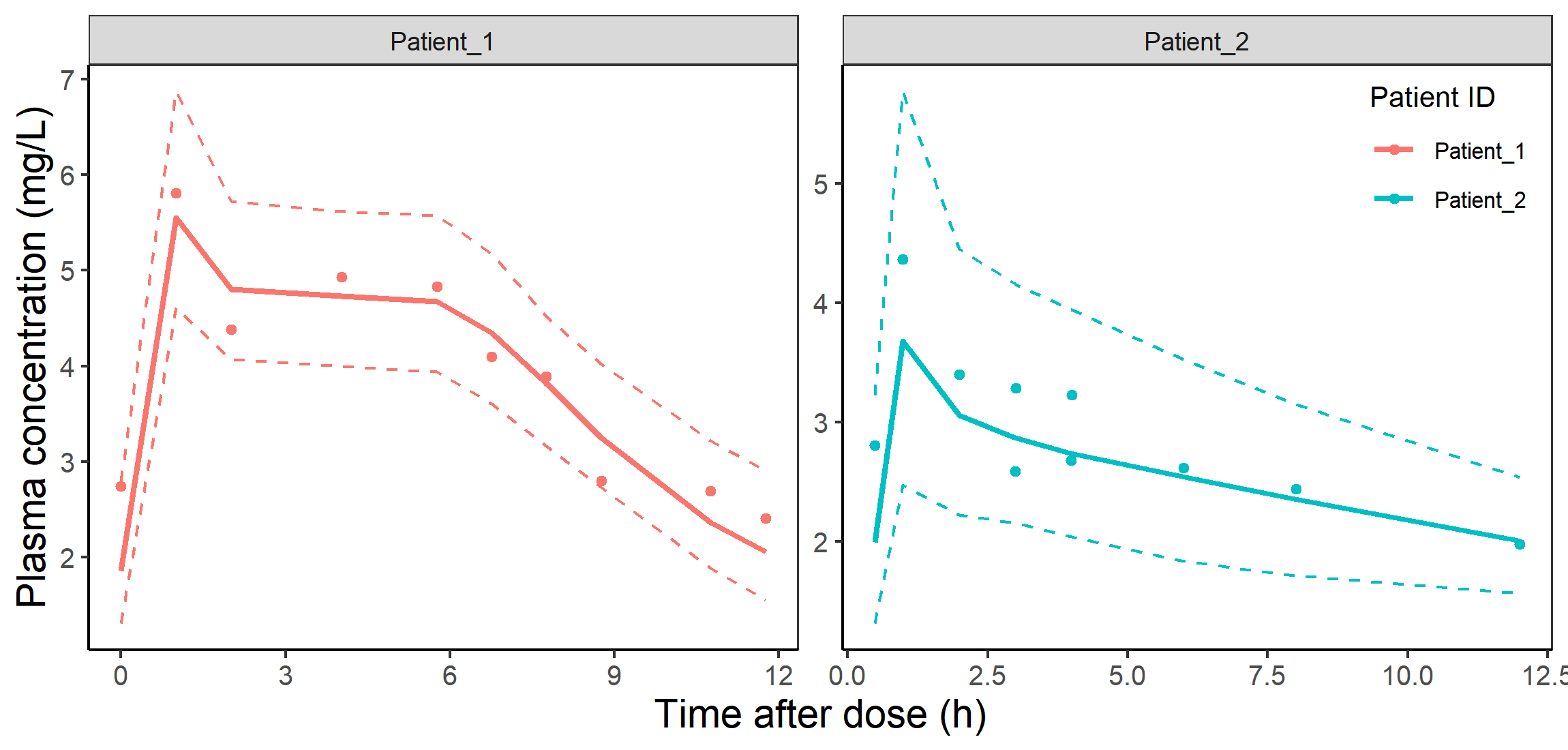


**Figure S5** Predicted versus observed values

The solid line indicates the median of the predicted values and the dashed line indicates the 90% reference range of the predicted values. Solid points indicate observed values.

**Section four: User guide**

**R Shiny Application for Fluconazole Dose Optimization in ARF Patients**

This software is an R Shiny application designed to optimize fluconazole dosing for patients with acute renal failure (ARF) undergoing renal replacement therapy (RRT), and can be freely accessible at <https://xy3yx.shinyapps.io/fluconazole-crrt-dosing/>. By inputting patient information, dosing details, and RRT-related data, the software can perform Monte Carlo simulations to predict plasma concentration-time curves and PK/PD outcomes. Below is the detailed user guide for the software.

**### Data Input Interface###**

The data input interface is organized using a drawer structure and includes the following modules:

**1. Patient and Dosing Information:**

**-** **Weight:** Enter the patient's weight (kg).

**-** **Loading Dose:** Enter the initial loading dose (mg).

**- Maintenance Dose: Enter the maintenance dose (mg).**

**- Dosing Start Time:** Click to enter the dosing start time (format: YYYY-MM-DD HH:MM).

**- Infusion Rate:** Enter the average infusion rate (mg/h).

**- Dosing Frequency and Interval:** Enter the frequency and interval of maintenance dosing (hours).

**2. RRT Information:**

**- Start Time:** Click to enter the start time for each RRT session (format: YYYY-MM-DD HH:MM).

**- End Time:** Click to enter the end time for each RRT session (format: YYYY-MM-DD HH:MM).

**- RRT Dose:** Enter the dose for each RRT session (the RRT dose is the sum of the dialysate flow rate (Q_D_) and the ultrafiltration rate (Q_UF_), mL/Kg/h).

**3. Dose Optimization:**

**- Supplemental Dosing Scenarios and Doses:** Select two supplemental dosing scenarios and enter the supplemental doses.

**- Scenario 1:** Supplement during each RRT session.

**- Scenario 2:** Supplement at the next dosing time after RRT ends.

**4. Simulation Information:**

**- Simulation Sample Size:** Enter the sample size for the simulation.

**- Minimum Inhibitory Concentration (MIC):** Enter the MIC (mg/L).

**- Observation Duration:** Enter the observation duration (hours).

**- Presenting Range:** Select the interval for presenting simulation results.

**### Result Output Interface###**

The result output interface provides real-time output and includes the following sections:

**1. Concentration-Time Curve Output:**

- Displays three concentration-time curves:

**- Black Curve:** Represents the dosing regimen without RRT.

**- Blue Curve:** Represents the dosing regimen with the specified RRT.

**- Green Curve:** Represents the dosing regimen with the specified RRT and supplemental dosing.

**- Pink Shaded Area:** Indicates the inputted RRT events.

**- Red Dashed Line:** Represents the reference concentration line at 11 μg/mL (research([Righi et al., 2019](#_ENREF_4)) shows that trough concentrations >11 μg/mL are significantly associated with clinical success, *p*=0.04).

**2. PK/PD Result Output:**

- Outputs *f*AUC/MIC values every 24 hours within the observation period.

**### Operational Steps###**

**1. Enter Patient and Dosing Information:**

- Fill in the respective fields with the patient's weight, loading dose, maintenance dose, dosing start time, infusion rate, and maintenance dose frequency and interval.

**2. Enter RRT Information:**

- Fill in the respective fields with the start time, end time, and dose for each RRT session.

**3. Set Dose Optimization Parameters:**

- Select the supplemental dosing scenarios and enter the corresponding supplemental doses.

**4. Enter Simulation Information:**

- Fill in the respective fields with the simulation sample size, MIC, observation duration, and reference range.

**5. Run the Simulation:**

- Click the **‘SIMULATION’** button. The application will perform Monte Carlo simulations to predict plasma concentration-time curves and PK/PD outcomes.

**6. View the Results:**

**- Concentration-Time Curves:**

- A) Assess the efficacy of the dosing regimen by observing the position of the three concentration-time curves relative to the red dashed line.

- B) Determine the necessity and effectiveness of supplemental dosing by comparing the green and blue curves relative to the red dashed line.

**- PK/PD Results:**

- C) Ensure that the 24-hour *f*AUC/MIC values are close to or exceed the target value (100 mg*h/L) to guarantee treatment efficacy.

This application provides clinicians with an efficient and flexible tool to optimize fluconazole dosing in complex clinical scenarios, ensuring patients receive the best possible therapeutic outcomes.

**Reference:**

AOKI, Y., TANIAI, N., YOSHIOKA, M., KAWANO, Y., SHIMIZU, T., KANDA, T., et al. (2018). Serum procalcitonin concentration within 2 days postoperatively accurately predicts outcome after liver resection. *Clin Chem Lab Med.* 56, 1362-1372. doi:10.1515/cclm-2018-0196

MUHL, E., MARTENS, T., IVEN, H., ROB, P. & BRUCH, H. P. (2000). Influence of continuous veno-venous haemodiafiltration and continuous veno-venous haemofiltration on the pharmacokinetics of fluconazole. *Eur J Clin Pharmacol.* 56, 671-8. doi:10.1007/s002280000216

PATEL, K., ROBERTS, J. A., LIPMAN, J., TETT, S. E., DELDOT, M. E. & KIRKPATRICK, C. M. (2011). Population pharmacokinetics of fluconazole in critically ill patients receiving continuous venovenous hemodiafiltration: using Monte Carlo simulations to predict doses for specified pharmacodynamic targets. *Antimicrob Agents Chemother.* 55, 5868-73. doi:10.1128/aac.00424-11

RIGHI, E., CARNELUTTI, A., BACCARANI, U., SARTOR, A., COJUTTI, P., BASSETTI, M., et al. (2019). Treatment of Candida infections with fluconazole in adult liver transplant recipients: Is TDM-guided dosing adaptation helpful? *Transpl Infect Dis.* 21, e13113. doi:10.1111/tid.13113

SINNOLLAREDDY, M. G., ROBERTS, M. S., LIPMAN, J., PEAKE, S. L. & ROBERTS, J. A. (2015a). Influence of sustained low-efficiency diafiltration (SLED-f) on interstitial fluid concentrations of fluconazole in a critically ill patient: Use of microdialysis. *Int J Antimicrob Agents.* 46, 121-4. doi:10.1016/j.ijantimicag.2015.02.017

SINNOLLAREDDY, M. G., ROBERTS, M. S., LIPMAN, J., ROBERTSON, T. A., PEAKE, S. L. & ROBERTS, J. A. (2015b). Pharmacokinetics of fluconazole in critically ill patients with acute kidney injury receiving sustained low-efficiency diafiltration. *Int J Antimicrob Agents.* 45, 192-5. doi:10.1016/j.ijantimicag.2014.08.013
